# The world needs BRICS countries to build capacity in invasion science

**DOI:** 10.1371/journal.pbio.3000404

**Published:** 2019-09-19

**Authors:** John Measey, Vernon Visser, Yury Dgebuadze, Inderjit, Bo Li, Michele Dechoum, Silvia R. Ziller, David M. Richardson

**Affiliations:** 1 Centre for Invasion Biology, Department of Botany and Zoology, Stellenbosch University, Matieland, South Africa; 2 Statistics in Ecology, Environment and Conservation, Department of Statistical Sciences, University of Cape Town, Rondebosch, Cape Town, South Africa; 3 African Climate and Development Initiative, University of Cape Town, Rondebosch, South Africa; 4 Severtsov Institute of Ecology and Evolution, Russian Academy of Sciences, Lomonosov Moscow State University, Moscow, Russia; 5 Department of Environmental Studies, University of Delhi, Delhi, India; 6 Ministry of Education Key Laboratory for Biodiversity Science and Ecological Engineering, Coastal Ecosystems Research Station of the Yangtze River Estuary, Institute of Biodiversity Science and Institute of Eco-Chongming, School of Life Sciences, Fudan University, Shanghai, China; 7 Departamento de Ecologia e Zoologia, Programa de pós graduação em Ecologia, Centro de Ciências Biológicas, Universidade Federal de Santa Catarina, Florianópolis, Santa Catarina, Brazil; 8 The Horus Institute for Environmental Conservation and Development, Florianopolis, Santa Catarina, Brazil

## Abstract

Developed countries are producing policies to reduce the flow of invasive species and control or eradicate existing invasions, but most developing countries are under-resourced to tackle either aspect without help. Emerging economies, such as Brazil, Russia, India, China, and South Africa (BRICS), are responsible for donating many of the world’s invasive species that have the potential to reach nearly all terrestrial biomes. Implementing a proactive ‘facilitated network’ model is urgently required to build capacity and stimulate effective appropriate invasion science. We contend that creating a BRICS network of invasion scientists will have the immediate impact required to meet future policy demands.

## Abstract 2

This Perspective article proposes that facilitated networks of invasion biologists in the BRICS countries (Brazil, Russia, India, China, South Africa) can quickly build the capacity needed to advance policy relating to invasive species.

## Introduction

All countries suffer from increasing problems with invasive species, but there is a divide between rich and poor nations in terms of progress in tackling this issue [1]. Developing countries are unlikely to meet Aichi Target 9 of the Convention on Biological Diversity (CBD) without help [2]. Emerging economies—such as Brazil, Russia, India, China, and South Africa (BRICS)—sit in between; they are experiencing increasing international and national trade but have limited capacity to translate research needed to inform relevant policy in their context. They are rapidly developing economies, but a large proportion of their populations have subsistence livelihoods although they have a growing footprint in both the developed and developing world. To this end, these countries have formed a forum (BRICS), primarily to discuss economics but also to interact on other issues of common interest. BRICS countries make up 26% of the terrestrial surface of the earth, have 42% of the planet’s human population and 14% of global GDP, and are home to a large proportion of the world’s biodiversity (e.g., the Brazilian Amazon, Cerrado, and Atlantic Forest; Russia’s Caucasus and Far East; Indian Western Ghats, Himalayas; Southwestern China; and South Africa’s Cape Floristic Region, Succulent Karoo, and Maputo-Pondoland-Albany). This biodiversity is under threat from anthropogenic drivers, including habitat conversion, exploitation, climate change, pollution, and species introductions [3]. Rapid economic growth in BRICS countries requires increasing trade, but not at the expense of their natural capital. This presents substantial challenges for legislation and enforcement in the absence of appropriate models in the developed or developing world.

One such challenge is invasive species, which were estimated to cost at least 0.1 billion US dollars (USD) per annum to the United States economy 2 decades ago [4], although a more recent annual cost of 1.7 billion USD to the UK economy [5] suggests that the current cost has escalated appreciably. The relationship between increasing economic activity and invasive species is well established [6], but even developed countries have been slow to implement and enact legislation curtailing the increasing effects of invasions. BRICS nations have growing trade both within and outside their borders, which is conducted at both large commercial and small artisanal scales. They are all signatories to the CBD and are thus currently preparing their responses to Aichi Target 9; these include the need to recognize invasive species, as well as determine their pathways of spread by 2020. Once the Aichi targets have been met, the CBD will set new targets relating to invasive species. Meeting new targets will require growing national capacity of invasion scientists with knowledge that relates to specific biomes within each BRICS nation. We posit that coordinating research and building capacity in invasion science deviates from a concentrated institute; instead, we propose the formation of a facilitated network of extant invasion biologists and social scientists with specialties across the biomes of BRICS countries. Furthermore, we present a model for this network and suggest how it could be implemented by BRICS countries to meet the next set of CBD targets in 2030.

## The invasion paradox in BRICS nations

**Fig 1:**
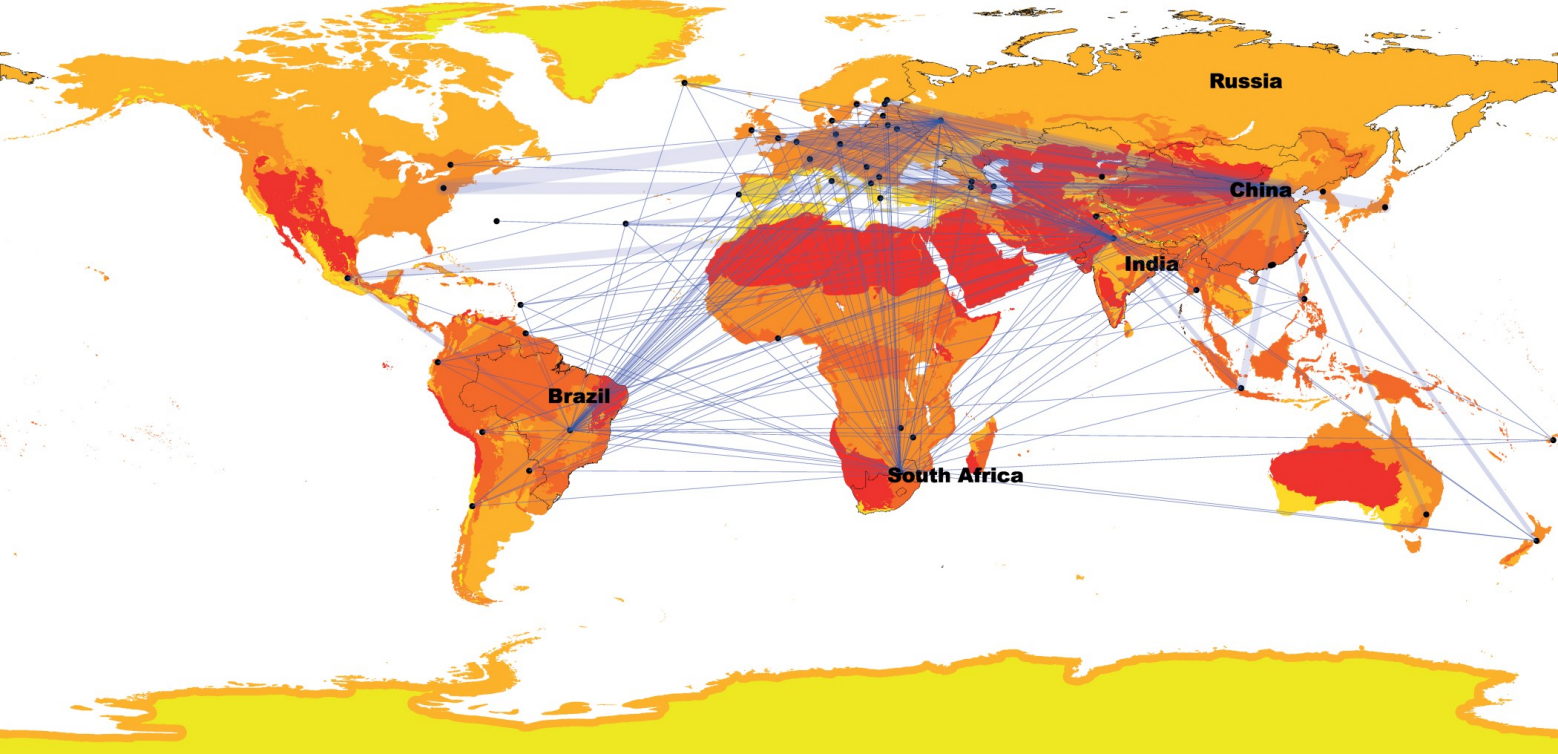
**When biomes from BRICS countries (labelled and with outlines) are projected onto the rest of the world, only the coldest areas are not represented by any biomes (green).** That so many of the world’s terrestrial areas are climate matched with BRICS countries suggests that many species from these emerging economies could become invasive elsewhere. Note that the colour scheme adds biomes that are present per BRICS country (and does not indicate different biomes), therefore the maximum number is 5. The volume of trade from BRICS countries (in USD) in 2018 is shown by the thickness of connecting lines (see S1 Text for details). BRICS, Brazil, Russia, India, China, and South Africa; USD, US dollars.

Unlike smaller, less diverse nations, BRICS countries suffer from invasive species that originate both within and outside of their borders. These ‘domestic exotic’ or ‘extra-limital’ invasions are especially relevant in large biodiverse countries such as the BRICS nations because there is often confusion regarding their status within the country [7] and consequently regulatory constraints. BRICS countries share invasive plant and animal species (Table 1A) and are the donors of some of the world’s most highly impacting species (Table 1B). Increasing trade from BRICS countries means that, unless unchecked, these areas are likely to become major donors of invasive species to the broader globe. Among the best predictors of invasive species are propagule pressure, commensurate with increasing trade, and climate suitability [8]. The diverse biomes and climatic conditions within the borders of BRICS countries suggest that they have suitable climatic diversity to cover all but the coldest biomes on Earth (Fig 1; S1–S5 Figs). Our analysis suggests that most of North America and Europe match climates in 3 or more biomes within BRICS countries but that Africa, South America, the Middle East, Asia, and Australia are matched by 4 or more biomes within BRICS countries. Interestingly, BRICS countries themselves have large areas with biomes that match each other. These commonalities among BRICS countries call for an interconnected facilitated network dealing with invasive species. In addition, there is increasing evidence that impacts of invasive species affect the poorest people in emerging economies worst [9], but in a world with a changing climate, effects of invasions have the potential to challenge sustainable economic development. For example, South Africa is estimated to have lost between 1.4 and 2.5 billion m^3^ of surface water runoff to invasive plant species, impacting drought-stricken cities like Cape Town [10].

**Table 1:** Some examples of the world’s worst invasive species that are (a) shared or (b) donated by BRICS countries. ‘N’ is native and ‘X’ is introduced; ‘X [N]’ indicates that the species is both native and introduced in different parts of each BRICS country.

Common Name	Species Name	Brazil	Russia	India	China	South Africa
**(a) Shared invasions**						
Black wattle	*Acacia mearnsii*	X		X	X	X
Giant African land snail	*Achatina fulica*	X		X	X	X
Chytrid fungus	*Batrachochytrium dendrobatidis*	X	X	X	X	X
Siam weed	*Chromolaena odorata*	X [N]		X	X	X
California scale	*Diaspidiotus perniciosus*	X	X	X	X [N]	X
Western mosquitofish	*Gambusia affinis*		X	X	X	X
Harlequin ladybird	*Harmonia axyridis*	X	X [N]		X [N]	X
Lantana	*Lantana camara*	X [N]		X	X	X
Leucaena	*Leucaena leucocephala*	X		X	X	X
American bullfrog	*Lithobates catesbeianus*	X			X	
Paperbark tree	*Melaleuca quinquenervia*				X	X
House mouse	*Mus musculus*	X	X	X	X	X
Rainbow trout	*Oncorhynchus mykiss*	X	X [N]	X	X	X
Mozambique tilapia	*Oreochromis mossambicus*	X		X	X	X [N]
Beach flea	*Platorchestia platensis*	X	X		X	X
Mesquite	*Prosopis juliflora*	X		X	X	X
Caster oil plant	*Ricinus communis*	X		X	X	X
Red-eared slider	*Trachemys scripta*	X	X		X	
Gorse	*Ulex europaeus*	X	X	X	X	X
**(b) Donated invasions**						
Common myna	*Acridotheres tristis*			X [N]	N	X
Tree of heaven	*Ailanthus altissima*			X	N	X
Asian long-horned beetle	*Anoplophora glabripennis*			X	N	
Giant reed	*Arundo donax*			N	N	X
Neem tree	*Azadirachta indica*			N	N	X
Ice plant	*Carpobrotus edulis*			X	X	N
House crow	*Corvus splendens*			X	X [N]	X [N]
Common carp	*Cyprinus carpio*	X	X [N]	X	N	X
Russian Olive	*Elaeagnus angustifolia*		N	N	N	
Argentine ant	*Linepithema humile*	N		X		X
Parthenium weed	*Parthenium hysterophorus*	N		X	X	X
Strawberry guava	*Psidium cattleianum*	N		X	X	X
Cane toad	*Rhinella marina*	N		X		
Giant salvinia	*Salvinia molesta*	N		X	X	X
Feral pig	*Sus scrofa*	X	X [N]	N	N	X
African clawed frog	*Xenopus laevis*				X	N

**Abbreviation:** BRICS, Brazil, Russia, India, China, and South Africa

## Facilitated networks in invasion science

The importance of global networks for invasion science has already been highlighted [11]. This underlines the urgency for focused translational research in invasion science, as well as the increase in power that results from a collaborative approach that extends the capacity of isolated research groups. Core projects undertaken by the group are complimented by satellite projects, which may result in joint capacity building and skills exchange between institutions and countries; successful examples of such ventures abound [11]. Equal to the requirement of rapid responses to research of emerging alien species is the need to substantially increase the capacity to tackle existing and future invasions in BRICS countries. Achieving this will require more than the transitory international collaborative projects advocated previously but rather in-country institutions that can maintain recruitment and extend beyond the careers of individual researchers, extending scientific knowledge to applied management carried out by government institutions and the nongovernmental organization (NGO) sector. While the standard approach to building institutions is to concentrate resources at a single location, we propose that ecological science demand a facilitated network approach to respond efficiently to invasion threats and to build and maintain capacity for the future.

The facilitated network revolves around a hub-and-spoke model drawing on existing excellence in invasion biology research within each country to grow capacity and collaboration over time. The hub (at the institution of the director) contains administrative staff to facilitate the network and disburse finances to Core Team Members (CTMs), already employed through their home institutions, and their associated researchers. While the hub may serve as a physical home representing the network, the network serves to study invasions in multiple contexts within the often-unique cultural and biological situations that exist elsewhere in the countries. Of key importance is the inclusion of social scientists alongside biologists; both economics and psychology are traditionally neglected fields when tackling problems—such as biological invasions—that are by definition linked to human activities and invoke complex human dimensions and the need for cultural changes. Annual research meetings bring all CTMs and students together in a conventional conference. Funding to have international plenaries present contextual, cutting-edge research should include representatives from other BRICS networks. Similarly, student awards should prioritize exchange between national BRICS meetings. Additionally, CTMs come together in a closed meeting at another time of year to discuss growth of the network and strategic directionality of research, including the planning of at least one themed workshop each year that should bring together selected CTMs and international participants to tackle emerging issues in invasion science. We suggest that within 5 years, a facilitated network of researchers in each of the BRICS countries will start making meaningful policy input as well as building capacity within their country and positively influencing their region. These facilitated networks are essentially Centres of Excellence (CoEs) that are distributed throughout the biomes of a country, with an administrative hub.

South Africa implemented the CoE model in 2004, to build excellence and capacity in nationally strategic research areas. The Centre of Excellence for Invasion Biology (CIB) was one of the first 5 funded CoEs and started with 14 CTMs in 4 of South Africa’s institutions [12]. The number of CTMs has grown to 27 in 9 institutions, covering the country’s 5 principal biomes as well as freshwater, marine, and terrestrial specialists. Their affiliations span universities, national parks, and government institutes and include staff from partner institutions embedded within the hub. The CIB was initially set up with 0.3 million USD and today receives 0.74 million USD but raises a further 52% (±19%) in co-funding. Of the total, 58.2% of funds are spent on student bursaries and running costs, producing 21 graduates annually in PhD (22%), MSc (35%), Honours (17%), and post doc students (22%). CIB alumni continue in academia (33.1%) or move into governmental and implementing agencies (17.2%), NGOs (5.5%), and other private sectors mostly relating to their fields of study. The CIB has contributed substantially to South African capacity in invasion science but has also had a significant effect on neighbouring countries through capacity development and studies. CTMs and their students and international associates now produce over 200 Web of Science (WoS)-listed publications annually, with more than a quarter in Q1 journals. Research outputs are well cited and respected. For example, almost 10% of references cited in Aichi Target 9 were published by the CIB [12]. Importantly, CTMs are responsible for facilitating production of government policy documents, and South Africa is the first country in the world to produce a national status report on biological invasions and their management [10], including the first framework of indicators for reporting on biological invasions at a country level [13].

While we actively advance this model for BRICS nations, there is no reason why many other countries, both developed and developing, should not adopt a similar model. However, the opportunities provided by the existing forum and agreements that are already in place for BRICS countries make it an attractive starting point. Many developing nations make significant advancements in research and implementation to counter the effects of invasive species [14]. Increased numbers of networks of researchers dedicated to invasion science will ultimately be beneficial to all [11].

## Toward a solution

The facilitated network approach proposed here offers many advantages to rapidly connect existing academics working on invasions to start building capacity and augment the research foundation on which national policy is formed. Firstly, each is already established and paid within their own institution and can offer biogeographic, cultural, and institutional insights from local invasions within their working context. By connecting these individuals through bi-annual meetings, granting opportunities, and shared bursaries, we expect meaningful collaborations on common problems to arrive intra- and inter-specifically. Capacity built by the networks can be rapidly absorbed into government and NGO sectors, and there will be an assured continuation of invasion biologists in academic positions. Once established, these networks can form cross-network links—building on the response of the global network on biological invasions—to positively influence the global response to invasions among developed and developing countries alike.

## Supporting information

S1 Methods Supplementary methods for modelling of climate matched areas of BRICS biomes, and the acquisition of trade data. References (Supplementary Only). BRICS, Brazil, Russia, India, China, and South Africa.
(DOCX)

S1 Text Description of biomes projected onto each of the BRICS nations. BRICS, Brazil, Russia, India, China, and South Africa.
(DOCX)

S1 Fig Biomes from Brazil projected onto the rest of the world. (TIF)

S2 Fig Biomes from Russia projected onto the rest of the world. (TIF)

S3 Fig Biomes from India projected onto the rest of the world. (TIF)

S4 Fig Biomes from China projected onto the rest of the world. (TIF)

S5 Fig Biomes from South Africa projected onto the rest of the world. (TIF)

## Abbreviations

BRICS: Brazil, Russia, India, China, and South Africa
CBD: Convention on Biological Diversity
CIB: Centre of Excellence for Invasion Biology
CoE: Centre of Excellence
CTM: Core Team Member
NGO: nongovernmental organisation
USD: US dollars
WoS: Web of Science

## References

[1] Seebens H, Blackburn TM, Dyer EE, Genovesi P, Hulme PE, Jeschke JM, et al No saturation in the accumulation of alien species worldwide. Nat Commun 2017;8: 14435 10.1038/ncomms14435 28198420 PMC5316856

[2] Nunez MA, Pauchard A Biological invasions in developing and developed countries: does one model fit all? Biol Invasions 2010;12: 707–714.

[3] IPBES Conceptual framework for the intergovernmental science-policy platform on biodiversity and ecosystem services (IPBES, Decision IPBES-2/4). 2013

[4] Pimentel D, Lach L, Zuniga R, Morrison D Environmental and economic costs of nonindigenous species in the United States. BioScience 2000;50: 53–66.

[5] Williams F, Eschen R, Harris A, Djeddour D, Pratt C, Shaw RS, et al The economic cost of invasive non-native species on Great Britain. CABI Proj No VM10066: 1–99. 2010

[6] Essl F, Dullinger S, Rabitsch W, Hulme PE, Hülber K, Jarošík V, et al Socioeconomic legacy yields an invasion debt. P Nat Acad Sci USA 2011; 108: 203–207. 10.1073/pnas.1011728108 PMC3017203 21173227

[7] Guo Q, Ricklefs RE Domestic exotics and the perception of invasibility. Divers Distrib 2010;16: 1034–1039.

[8] Hayes KR, Barry SC Are there any consistent predictors of invasion success? Biol Invasions 2008;10: 483–506.

[9] Shackleton CM, McGarry D, Fourie S, Gambiza J, Shackleton SE, Fabricius C Assessing the effects of invasive alien species on rural livelihoods: case examples and a framework from South Africa. Hum Ecol 2007;35: 113–127.

[10] van Wilgen BW, Wilson JR, editors The status of biological invasions and their management in South Africa. Pretoria: Government of South Africa. 2018.

[11] Packer JG, Meyerson LA, Richardson DM, Brundu G, Allen WJ, Bhattarai GP, et al Global networks for invasion science: benefits, challenges and guidelines. Biol Invasions 2017; 19: 1081–1096.

[12] van Wilgen BW, Davies SJ, Richardson DM Invasion science for society: A decade of contributions from the Centre for Invasion Biology. S Afr J Sci 2014; 110: 1–12.

[13] Wilson JR, Faulkner KT, Rahlao SJ, Richardson DM, Zengeya TA, van Wilgen BW Indicators for monitoring biological invasions at a national level. J Appl Ecol 2018; 55: 2612–2620.

[14] Zenni RD, Ziller SR, Pauchard A, Rodriguez-Cabal M, Nuñez MA Invasion science in the developing world: A response to Ricciardi et al Trends Ecol Evol 2017; 32: 807–808. 10.1016/j.tree.2017.08.006 28867137

