# Supplementary material for: The world needs BRICS countries to build capacity in invasion science
Source: PLoS Biol. 2019 Sep 19;17(9):e3000404. doi: 10.1371/journal.pbio.3000404 (PMC6772094; doi:10.1371/journal.pbio.3000404)
Supplement: S1 Text — BRICS, Brazil, Russia, India, China, and South Africa. (DOCX) [file pbio.3000404.s002.docx]

**S1 Text**

**Supplementary Methods:**

Modelling climate matched areas

We used the 14 terrestrial ecoregions of the world as defined by Olson et al. (2001) to determine the areas of the globe climatically matched to biomes present in Brazil, Russia, India, China and South Africa (BRICS). These ecoregions capture major variations in global climate and disturbance regimes. We acknowledge that this level of climate matching is relatively coarse, yet the similarity of areas such as Mediterranean ecosystems have formed the basis of much work in biological invasions (e.g. Groves & Di Castri 1991). This produced a candidate list of biomes distributed globally for each of the BRICS countries (Figs. S1 to S5). We then added each of these layers together to produce a sum of all biomes represented by BRICS countries globally (Fig. 1).

Trade data

We used the UN Comtrade Database (available from http://www.hostgis.com/datasources/pages/international/comtrade.php) to generate global trade data for exports from BRICS countries to the rest of the world during 2018. We selected commodity only trade (excluding services as these are rarely considered to be pathways for invasive species: Wilson et al. 2009). Only the BRICS countries (Brazil, Russian Federation, India, China [China, Hong Kong SAR; China, Macao SAR] and South Africa) were selected as reporters (exporting country), while all countries (world) were considered as partners (importing countries). We included all Harmonized System (HS) commodities in the dataset, as although invasive species are more likely to be moved with (for example) timber exports, any containers and their vessels traded are potential carriers of alien propagules (Wilson et al. 2009). As trade-flows, only exports were included in the dataset. This provided us with a dataset of exports of goods from BRICS countries to the rest of the world quantified in US$.

Each trading partnership reported was summed and this was divided into 6 bins using the ‘natural breaks [Jerks]’ function in QGIS (QGIS Development Team 2019). Each bin was given a different thickness representing the level of trade as follows:

| **Trade level in US$** |
| --- |
| 0 – 84,853,182 |
| 84,853,182 – 298,955,600 |
| 298,955,600 – 645,591,874 |
| 645,591,874 – 1,001,141,522 |
| 1,001,141,522 – 1,599,878,197 |
| 1,599,878,197 – 2,146,323,000 |

**S1-S5 Fig**

When biomes from BRICS (Brazil, Russia, India, China and South Africa) countries are projected onto the rest of the world, only the coldest areas are not represented by at least one biome (grey). Here, the biomes of each BRICS country are projected individually onto the rest of the earth.

**References**

Groves, R.H., Di Castri, F. and Groves, R.H. eds., 1991. Biogeography of Mediterranean Invasions. Cambridge University Press.

Olson, D.M., Dinerstein, E., Wikramanayake, E.D., Burgess, N.D., Powell, G.V., Underwood, E.C., D'amico, J.A., Itoua, I., Strand, H.E., Morrison, J.C. and Loucks, C.J., 2001. Terrestrial Ecoregions of the World: A New Map of Life on EarthA new global map of terrestrial ecoregions provides an innovative tool for conserving biodiversity. BioScience, 51(11), 933-938.

QGIS Development Team (2019). QGIS Geographic Information System. Open Source Geospatial Foundation Project. http://qgis.osgeo.org

Wilson, J.R., Dormontt, E.E., Prentis, P.J., Lowe, A.J. and Richardson, D.M., 2009. Something in the way you move: dispersal pathways affect invasion success. Trends in ecology & evolution, 24(3),136-144.
