# Supplementary figures and images for: The world needs BRICS countries to build capacity in invasion science
Source: PLoS Biol. 2019 Sep 19;17(9):e3000404. doi: 10.1371/journal.pbio.3000404 (PMC6772094; doi:10.1371/journal.pbio.3000404)

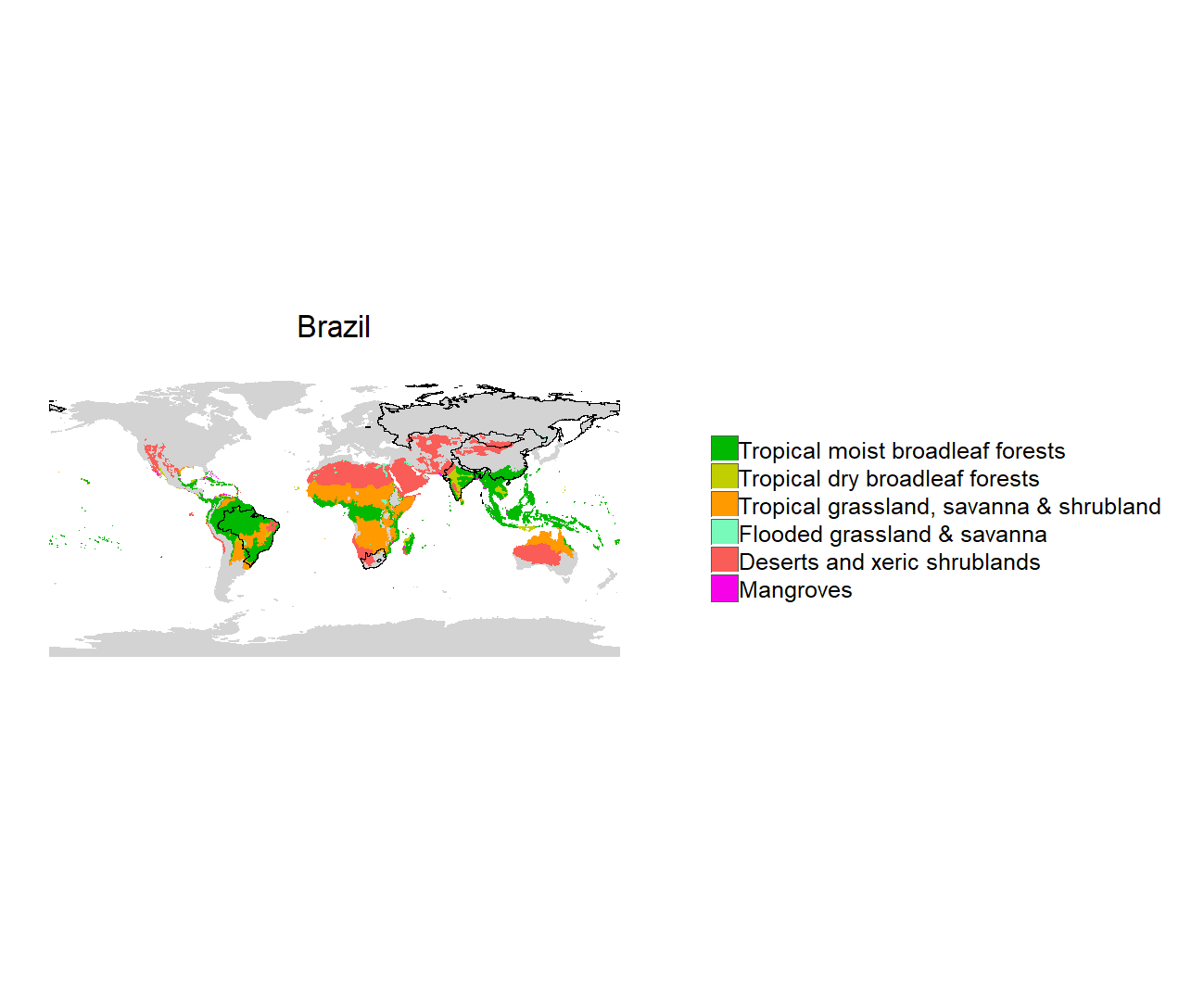

Supplement: S1 Fig — (TIF) [file pbio.3000404.s003.tif]

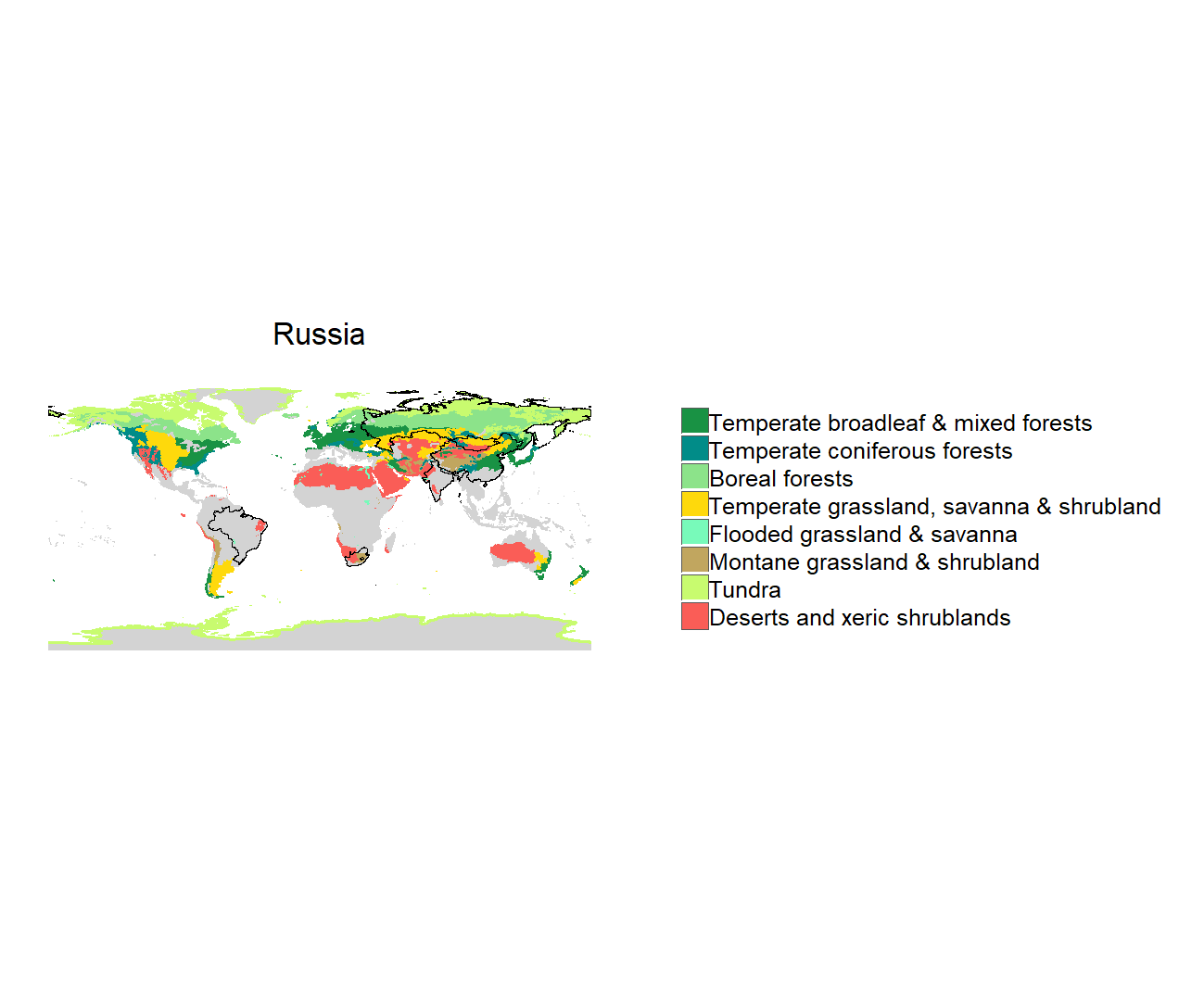

Supplement: S2 Fig — (TIF) [file pbio.3000404.s004.tif]

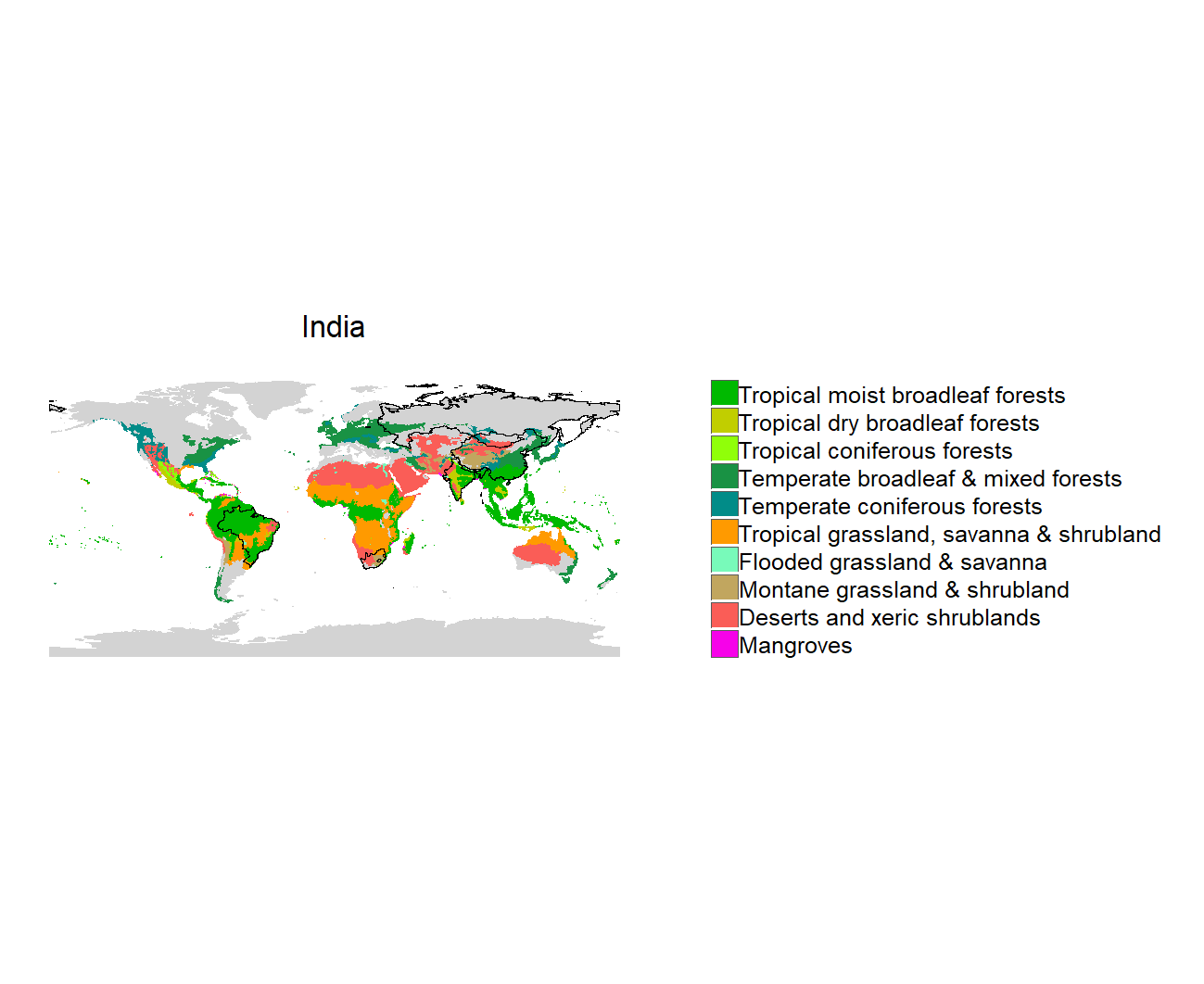

Supplement: S3 Fig — (TIF) [file pbio.3000404.s005.tif]

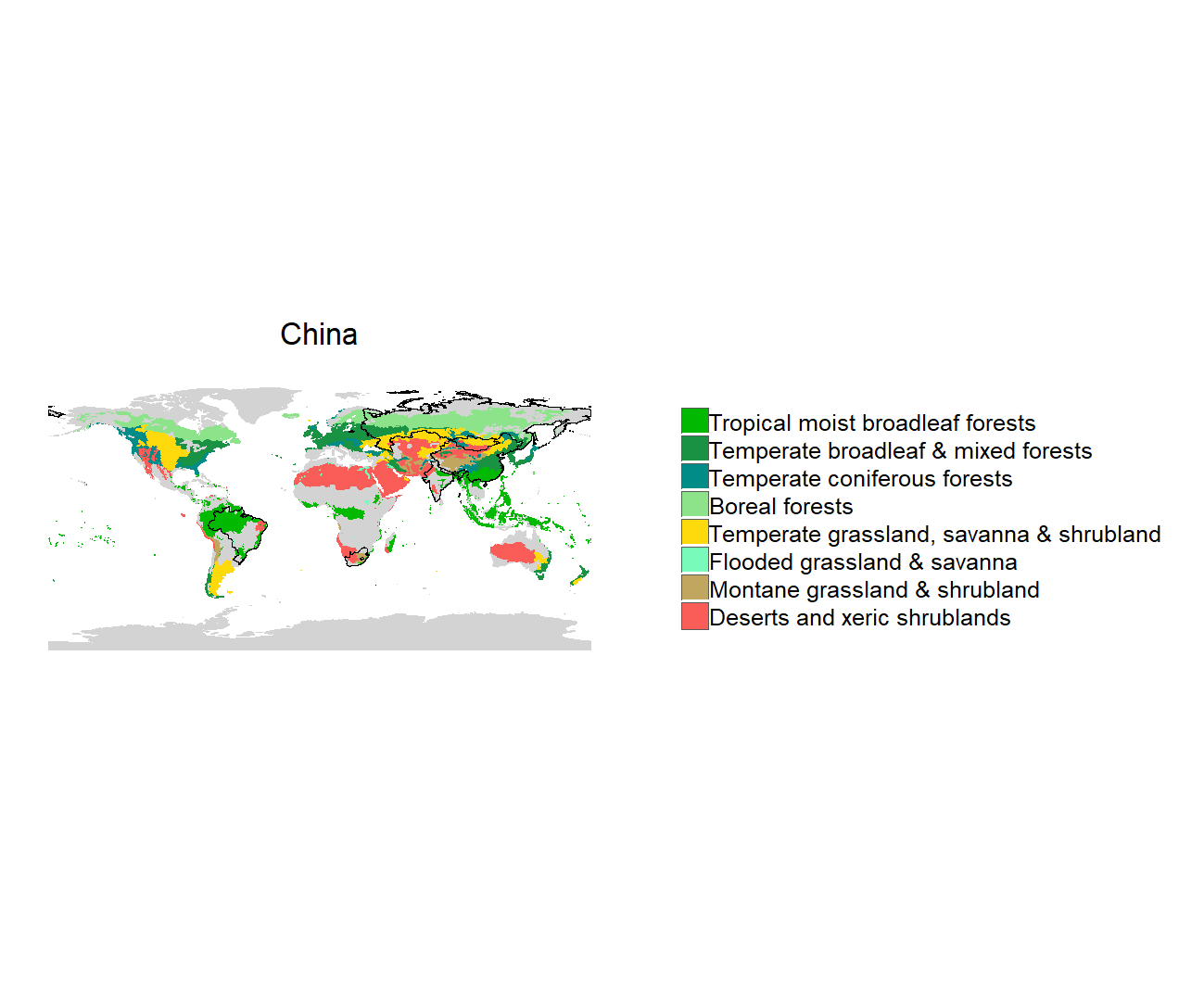

Supplement: S4 Fig — (TIF) [file pbio.3000404.s006.tif]

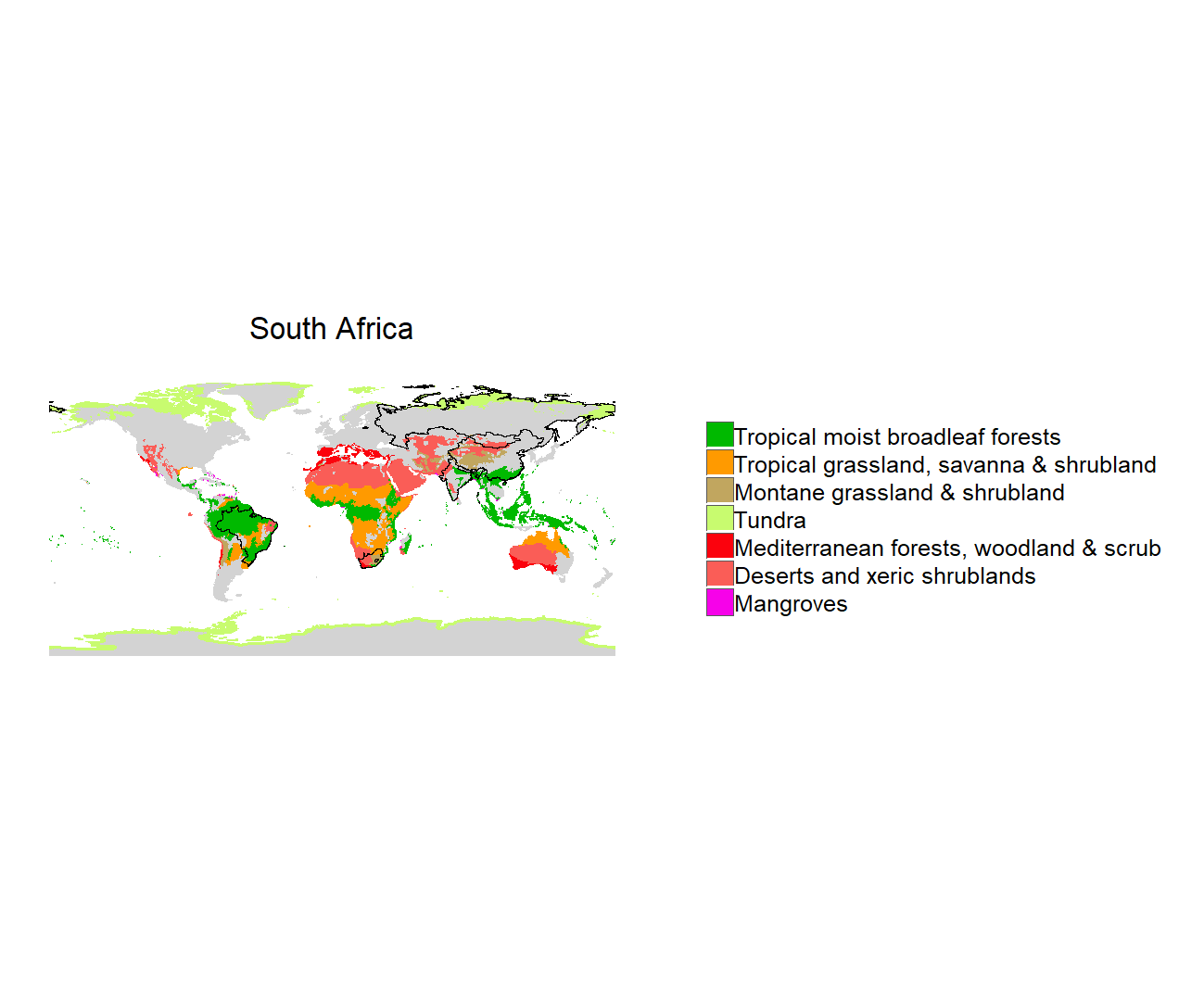

Supplement: S5 Fig — (TIF) [file pbio.3000404.s007.tif]
